# Virtual Endocrinology Care Emphasizing Data-Driven Insights and Continuous Engagement and Its Impact on Glycemic Outcomes in Patients With Uncontrolled Diabetes: A Real-world Retrospective Case Series

**DOI:** 10.2196/30626

**Published:** 2022-03-24

**Authors:** Calvin C Wu, Karin C Wu, Aimée S José, Niloufar Novin

**Affiliations:** 1 Steady Health, Inc San Francisco, CA United States; 2 Endocrine Research Unit San Francisco Veterans Affairs Health Care System San Francisco, CA United States

**Keywords:** continuous glucose monitoring, connected care, digital health, telemedicine, type 1 diabetes, type 2 diabetes

## Abstract

**Background:**

Steady Health’s novel virtual care model incorporates continuous glucose monitoring (CGM) and a multidisciplinary approach to timely person-centered diabetes care.

**Objective:**

This real-world retrospective case series explores the early glycemic outcomes of its patients with uncontrolled diabetes.

**Methods:**

All patients of Steady Health who had an initial time in range (TIR) below 70% from their first 4 weeks of available CGM data and who had completed onboarding by February 2021 were included in this analysis. We compared the change in TIR, time below range, and average blood glucose from their first 4 weeks with their latest 4 weeks of available CGM data. Hemoglobin A_1c_ (HbA_1c_) values at baseline and at the end of the study were also compared. Patients completed a questionnaire assessing their satisfaction with Steady Health’s intervention.

**Results:**

A total of 53 patients (n=35, 66% with type 1 diabetes; n=44, 83% treated with insulin) were included in this analysis. This cohort had a median baseline TIR of 53.0% (IQR 40.9%, 61.7%) and saw a median change in TIR of +16.6% (IQR +6.0%, +27.9%; *P*<.001) over a median duration of care of 11 months, amounting to nearly 4 more hours spent between 70 to 180 mg/dL a day. Of the 27 patients who had both baseline and follow-up HbA_1c_ results, their median baseline HbA_1c_ was 8.6% (IQR 7.5%, 11.4%; 70 mmol/mol), while their median change in HbA_1c_ was –1.2% (IQR –2.6%, –0.2%; *P*=.001). Importantly, these glycemic improvements were achieved with a median decrease in the time below range by –0.3% (IQR –1.1%, 0.0%; *P*<.001), regardless of whether patients were started on an automated insulin delivery system. A total of 40 (75.5%) patients improved TIR by ≥5%, and 27 (50.9%) achieved TIR≥70% by the end of the study. Glycemic improvements were greatest among patients with the lowest baseline TIR and those who collaborated most intensively with Steady Health’s clinicians. A total of 25 of these patients responded to a questionnaire assessing levels of satisfaction with their care, and all of them agreed that Steady Health had a positive impact on their diabetes management.

**Conclusions:**

Our findings suggest that patients with uncontrolled diabetes can achieve significant glycemic improvements by working with a virtual multidisciplinary care team that uses CGM to provide continuous clinical feedback and support.

## Introduction

Diabetes currently affects more than 10% of the US population [1], and its prevalence is projected to double by 2060 [2]. As the seventh leading cause of death in the United States [3] and a major driver of cardiovascular disease, kidney failure, and blindness, diabetes imposes the greatest economic burden of any chronic condition [4,5], to say nothing of its immeasurable impact on quality of life and well-being. Even though optimal glycemic control can prevent and delay diabetes complications, only about half of US adults with diabetes meet recommended hemoglobin A_1c_ (HbA_1c_) treatment targets [6].

Unfortunately, current treatment paradigms fail to meet the complex and dynamic demands of this unrelenting disease. People with diabetes make daily, even hourly, decisions to manage their condition, but they are poorly supported by the conventional care model that consists of 1 to 4 visits a year with a health care provider. Limited time and resources prevent most providers from reviewing more than a HbA_1c_ level and a summary of blood glucose (BG) data, and opportunities to address mental health and promote effective lifestyle modification are often missed. Moreover, access to specialized care remains restricted amid a substantial shortage of diabetes providers, with patients with diabetes outnumbering diabetes educators by more than 1600:1 [7] and three-quarters of US counties not having an endocrinologist [8].

Meanwhile, technologies and therapies aimed at improving diabetes care are advancing at a substantial pace. We have more treatment options than ever before: medications targeting specific pathophysiological defects, both faster- and longer-acting insulins, and automated insulin delivery systems. Continuous glucose monitoring (CGM) deserves special mention as an excellent complement to HbA_1c_ when assessing glycemic control. By capturing a complete BG profile throughout the day, CGM can more immediately inform treatment decisions and lifestyle modifications. Unfortunately, these advances can improve outcomes only if they reach the patients with diabetes who need them. Many providers struggle to keep up with the rapidly changing landscape of diabetes treatment options. Other complicating factors include high treatment costs, lack of access to care, therapeutic inertia, and inadequate patient education and support. Indeed, ongoing efforts have yet to translate into better clinical outcomes as the percentage of patients with diabetes achieving glycemic targets remains stagnant over time [6,9].

There are no neat solutions that overcome all these challenges at once, but we believe that the way we deliver care to people with uncontrolled diabetes needs an overhaul. Patients with diabetes benefit from a comprehensive assessment by an endocrinologist, who can identify gaps and deficiencies in diabetes self-management, connect patients with diabetes with the best tools and resources, and put in motion a care plan tailored to their specific needs. Diabetes care and education specialists (DCESs) can provide essential education to patients with diabetes, train them to use new technology, coach them on nutrition and exercise, and answer day-to-day questions regarding diabetes management. Geographic barriers are minimized, and multidisciplinary care becomes convenient and continual when it is provided over telemedicine and messaging. CGM data can be remotely monitored and analyzed alongside details of the patient’s lifestyle, and insights derived from that data can foster learning and self-improvement, and guide timely clinical interventions. We present a novel virtual diabetes care model that incorporates all these components and explore its early efficacy in patients with uncontrolled diabetes.

## Methods

### Ethical Considerations

The study was evaluated by the Advarra Institutional Review Board (Pro00061557) and approved for exemption from IRB oversight. All patient data was anonymized prior to analysis and no identifiable protected health information was included in this publication.

### Steady Health

Steady Health is a virtual endocrinology clinic currently available to patients with diabetes in California and Washington (with plans to expand nationwide), with a monthly membership fee. Steady Health leverages CGM and telemedicine to provide personalized, data-driven care and features a multidisciplinary team approach led by endocrinologists, DCESs, and care coordinators.

All care is provided exclusively through the Steady Health app. Patients can exchange messages (responses are provided within 24 hours on weekdays) and schedule telemedicine visits with their care team within a few days. As a data collection tool, the Steady app allows for sharing of meals, insulin dosing, exercise, notes, and CGM data. These inputs are consolidated into a single view within the Steady Health software platform for clinicians to analyze in detail. Patients can view and learn from the BG profile associated with each logged event in the app, as well as access their clinical reports and visit note summaries.

### Onboarding and Engagement

The onboarding process involves two visits with an endocrinologist and a “tracking period” in between. The first visit is an opportunity to gather and thoroughly understand the patient’s medical history, which serves as the foundation for future care; the tracking period meticulously explores how the patient manages their diabetes day to day; and the tracking review visit takes the form of an open discussion, in which insights are highlighted and used to craft a long-term care plan.

During the first visit, a routine assessment is performed, with added emphasis on the patient’s mental health and current struggles. Patients are asked to complete the Problem Areas in Diabetes questionnaire, and their responses are reviewed during the visit; if the patient agrees they might benefit from working with a mental health professional, a referral is made to one of several partnering psychologists specializing in diabetes distress. If patients are not already using one, they are prescribed a CGM and instructed on how to use the device and interpret readings. Data from Dexcom Clarity are obtained via a web application programming interface, whereas the raw data from LibreView and CareLink are periodically downloaded then uploaded to Steady Health’s software platform. Once CGM use is initiated and BG data are shared wirelessly, patients complete a 7-day tracking period. This includes photo journaling their meals and logging their exercise, insulin, and notes, which together with their BG data establish a diagnostic baseline of their diabetes management. Patients then have a 1-hour follow-up visit with their endocrinologist to review learnings from their tracking period and set personalized goals and projects to improve their diabetes management.

As part of ongoing care, DCESs provide education and coaching tailored to each patient’s needs and schedule. Patients receive a monthly report from their endocrinologist that includes a summary of their BG statistics and a personalized message reviewing their progress. Patients may also receive biweekly notifications if they meet or dip below their self-identified time in range (TIR) goals. These built-in touch points allow Steady Health to proactively reach out and continuously engage with patients about their diabetes.

Steady Health’s care model allows clinicians to start patients with diabetes on CGM, smart pens, and pumps completely remotely. Steady Health’s clinicians have a deep knowledge of the latest diabetes advancements and recommend the best tools for meeting each patient’s unique needs. Although the training for these devices is conventionally provided in person, Steady Health has developed online instructional material and offers one-on-one video appointments to ensure a smooth transition.

With greater shared insight into BG and lifestyle data, Steady Health empowers patients with a deeper understanding of the factors that impact BG, so they may play a more active role in their diabetes management. As they work with patients on educational topics, medication adjustments, and behavioral modification, Steady Health’s clinicians emphasize meeting patients where they are in their journey, rather than going through a prescribed program. The clinic has a general framework for onboarding and follow-up, but patients have personalized plans for improvement projects and can engage with their clinicians as often as they like.

### Study Design and Participants

This study was conducted in accordance with the Helsinki Declaration. A database review was performed in February 2021 to identify all Steady Health patients with uncontrolled diabetes who had completed onboarding. Patients came to the clinic either through the website or word of mouth, or they were referred by their health care provider between October 2019 and December 2020. Patients with uncontrolled diabetes were defined as having a TIR below 70% during their first 4 weeks of available CGM data. International consensus describes TIR as the percentage of time spent within a target BG range of 70 to 180 mg/dL and recommends a goal TIR>70% for patients with type 1 and type 2 diabetes [10]. Several studies suggest an association between TIR and the risk for microvascular complications [11-15].

For patients meeting inclusion criteria, the TIR, time below range (TBR), and average BG from their first 4 weeks of available CGM data were collected and used to define their baseline glycemic control. TBR refers to the percentage of time spent below 70 mg/dL, and international consensus recommends a goal TBR<4% for patients with type 1 and type 2 diabetes [10]. As a comparison to evaluate the impact of Steady Health’s intervention, TIR, TBR, and average BG from the latest 4 weeks of available CGM data were used to quantify glycemic control at the study’s end. Demographic characteristics and clinical data, including HbA_1c_ results, were obtained by a review of the medical record.

Because many patients were able to achieve and maintain glycemic improvements after a focused but variable period of active engagement with their care team (often resulting in lasting changes in lifestyle or therapy), we chose to focus on the intensity of these interactions. Maximal engagement was therefore quantitatively assessed by the number of clinically relevant encounters or messages exchanged over a period of 4 weeks at any point during each patient’s care, and categorized as high (≥10), moderate (5-9), or low (<5).

Lastly, to assess levels of satisfaction with Steady Health’s role in their ongoing diabetes care, an anonymous 9-question questionnaire (using a 5-point Likert scale) was sent to these 53 patients.

### Statistical Analysis

Baseline characteristics of participants were presented using means and SDs or medians and IQRs for continuous measures (depending on normality) and counts and percentages for categorical measures. Absolute change in the outcome measures (TIR, TBR, HbA_1c_, average BG) were reported as median and IQR given skewed distributions, and the Wilcoxon signed rank tests were used to determine whether study outcomes changed between enrollment and the study’s end. Linear regression models were used to determine the associations between levels of maximal engagement and absolute change in TIR, TBR, HbA_1c_, and average BG, adjusted for age, sex, diabetes type, and insulin type. Covariates were selected a priori based on the understanding of the causal network relating treatment to outcome. The appropriateness of treating levels of maximal engagement as a continuous variable (as opposed to an ordinal variable) was assessed with likelihood ratio tests for all models. All analyses were performed with STATA 15.1 software (StataCorp).

## Results

A total of 53 patients met inclusion criteria and are described in Table 1. The mean duration of care was 11 (range 3-27) months.

**Table 1:** Baseline characteristics.

		Patients (N=53)
Age (year), mean (SD)		39.8 (11.7)
Female, n (%)		24 (45.3)
**Ethnicity, n (%)**		
	Caucasian	36 (67.9)
	Asian	8 (15.1)
	Hispanic/Latinx	6 (11.3)
	African American	3 (5.7)
**Diabetes type, n (%)**		
	Type 1	35 (66.0)
	Type 2	18 (34.0)
Commercially insured, n (%)		52 (98.1)
**Complications, n (%)**		20 (37.7)
	Peripheral neuropathy	10 (18.9)
	Diabetic retinopathy	11 (20.8)
	Nephropathy	7 (13.2)
	Cardiovascular disease	1 (1.9)
Duration of care (months), median (IQR)		11 (5, 18)
**HbA_1c_^a^ (%; n=41), median (IQR)**		8.5 (7.5, 11.2)
	<8%, n (%)	17 (41.5)
	8%-10%, n (%)	10 (24.4)
	>10%, n (%)	14 (34.1)
**Time in range^b^ (%), median (IQR)**		53.0 (40.9, 61.7)
	0%-30%, n (%)	11 (20.8)
	30%-50%, n (%)	12 (22.6)
	50%-60%, n (%)	13 (24.5)
	60%-70%, n (%)	17 (32.1)
**Time below range^c^ (%), median (IQR)**		0.9 (0.2, 2.5)
	0%-1%, n (%)	28 (52.8)
	1%-4%, n (%)	18 (34.0)
	>4%, n (%)	7 (13.2)
**Treatment with insulin, n (%)**		44 (83.0)
	Injection	25 (56.8)
	Pump	19 (43.2)

^a^HBA_1c_: hemoglobin A_1c_.

^b^Time in range defined as % of time glucose falls between 70-180 mg/dL over 28 days.

^c^Time below range defined as % of time glucose falls below 70 mg/dL over 28 days.

### Improvements in Glycemic Outcomes

**Figure 1:**
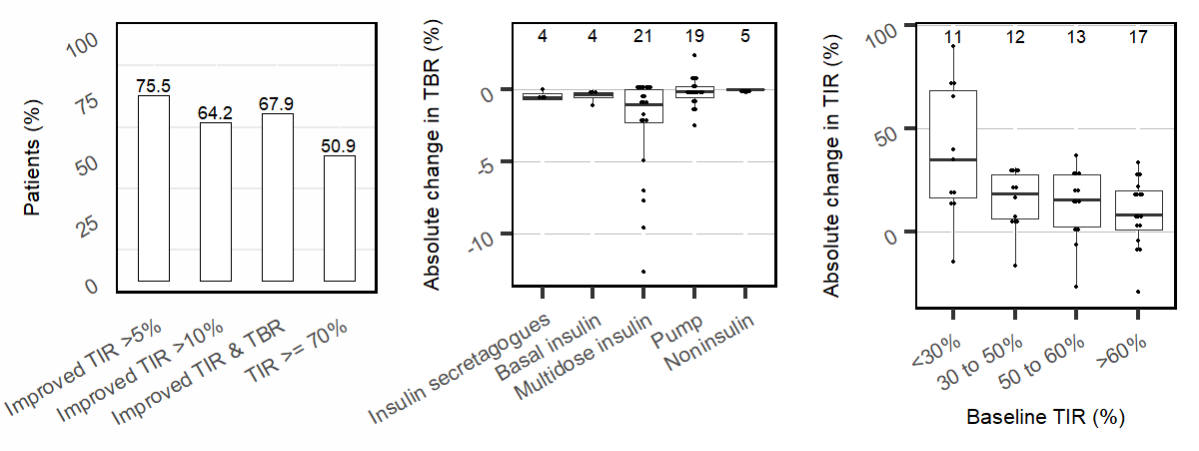
The percentage of patients in this cohort who improved TIR by ≥5%, improved TIR by ≥10%, simultaneously improved TIR while maintaining or lowering TBR, and achieved TIR≥70% by end of the study are shown in the graph on the left. The absolute change in TBR by type of therapy (insulin secretagogues, basal insulin injections only, multiple daily insulin injections, insulin pump, and no insulin use whatsoever) is shown in the middle graph. The absolute change in TIR based on the baseline TIR category is shown in the graph on the right. All box plots are shown with median, IQR, and individual data points. The number on top in the middle and right graphs denote the number of patients in each category described. TBR: time below range; TIR: time in range.

Initial values of TIR, TBR, HbA_1c_, and average BG, and the absolute change in each glycemic metric by the end of study are shown in Table 2. Comparing glycemic parameters at baseline and at the end of the study, 40 (75.5%) patients increased their TIR by at least 5%, and 34 (64.2%) improved their TIR by 10% or more. Meanwhile, 36 (67.9%) patients simultaneously increased their TIR while maintaining or reducing their TBR, and 27 (50.9%) achieved TIR of 70% or greater (Figure 1, left). Although 7 patients had an initial TBR exceeding 4%, only 2 of these had a TBR greater than 4% at the end of the study. The greatest reduction in TBR was seen among patients on multiple daily insulin injections (Figure 1, middle).

**Table 2:** Glycemic outcomes.

	Initial values, median (IQR)^a^	∆ (end of study), median (IQR)^a^	*P* value
Time in range (%)	53.0 (40.9, 61.7)	+16.6 (+6.0, +27.9)	<.001
Time below range (%)	0.9 (0.2, 2.5)	–0.3 (–1.1, 0.0)	<.001
HbA_1c_^b^ (%; n=27)	8.6 (7.5, 11.4)	–1.2 (–2.6, –0.2)	.001
Average glucose (mg/dL)	180 (163, 193)	–21 (–34, –6)	<.001

^a^Values are reported as medians (IQR) for these outcomes with skewed distribution. Wilcoxon signed rank tests were used.

^b^HbA_1c_: hemoglobin A_1c_.

### Change in TIR by Baseline TIR Category

There was a progressively greater increase in TIR with lower baseline TIR (Figure 1, right). Those patients with a baseline TIR below 30% had the greatest increase in TIR of 35.1% (IQR 16.8%, 68.4%), equating to 8.4 additional hours spent in range, while those patients with a baseline TIR of 60.1%-69.9% had an increase in TIR of 8.2% (IQR 1.1%, 19.8%).

### Remote Initiation of Diabetes Treatments

Of the 53 patients, 20 (37.7%) transitioned to either a new insulin pump or algorithm, 4 (7.5%) began using a smart pen, and 3 (5.7%) initiated inhaled insulin during their care. Of these 27 patients who successfully switched to a new mode of insulin delivery, 23 (85.2%) saw improvements in their TIR, and 2 more who had been experiencing excess hypoglycemia meaningfully reduced their TBR by more than 2.1% or 30 minutes a day. In addition, we performed a sensitivity analysis to examine the outcomes in those who transitioned to an automated insulin delivery system (17/53 patients) during this study and those who did not (36/53 patients). We found that the absolute changes in TIR, TBR, HbA_1c_, and average BG did not differ significantly between these two groups.

### Glycemic Improvements by Level of Maximal Engagement

Greater levels of maximal engagement were associated with more substantial reductions in HbA_1c_ and average BG, and increases in TIR, without an increase in TBR (Tables 3 and 4). These relationships strengthened with the adjustment for age, sex, diabetes type, and type of insulin therapy. For every level up in maximal engagement, there was an average increase in TIR by 13.5% (adjusted *P*=.01) and an average decrease in HbA_1c_ by 1.3% (adjusted *P*=.03).

**Table 3:** Levels of maximal engagement and changes in glycemic outcomes (simple linear regression).

			Coefficients^a^ (simple linear regression)														
			∆ TIR^b^ (%)		*P* value		∆ TBR^c^ (%)		*P* value		∆ A_1c_^d^ (%)		*P* value		∆ aBG^e^ (mg/dL)		*P* value
**Maximal engagement**			+8.61		.04		–0.00		.99		–0.97		.06		–17.50		.01
	*R* ^2^	0.08				0.00				0.14				0.11			
	*F* (*df*)	4.33 (1,51)				0.00 (1,51)				3.83 (1,24)				6.39 (1,51)			

^a^Coefficients referred to a change in outcome per level up in maximal engagement (low, medium, high).

^b^TIR: time in range.

^c^TBR: time below range.

^d^A_1c_: hemoglobin A_1c_.

^e^aBG: average blood glucose.

**Table 4:** Levels of maximal engagement and changes in glycemic outcomes (multiple linear regression).

			Coefficients^a^ (multiple linear regression^b^)														
			∆ TIR^c^ (%)		*P* value		∆ TBR^d^ (%)		*P* value		∆ A_1c_^e^ (%)		*P* value		∆ aBG^f^ (mg/dL)		*P* value
**Maximal engagement**			+13.51		.002		–0.15		.77		–1.34		.01		–25.16		.001
	*R* ^2^	0.36		.01		0.23		.13		0.59		.03		0.38		.005	
	*F* (*df*)	3.16 (8,44)				1.66 (8,44)				3.01 (8,17)				3.34 (8,44)			

^a^Coefficients referred to a change in outcome per level up in maximal engagement (low, medium, high).

^b^Multiple linear regression model adjusted for age, sex, diabetes type, and insulin type.

^c^TIR: time in range.

^d^TBR: time below range.

^e^A_1c_: hemoglobin A_1c_.

^f^aBG: average blood glucose.

### Measures of Patient Satisfaction

Of the 53 patients surveyed, 25 (47.2%) responded. Of these 25 respondents, 23 (92%) strongly agreed and 2 (8%) agreed that Steady Health had a positive impact on their diabetes management. Meanwhile, 18 (72%) respondents strongly agreed and 5 (20%) agreed that they felt supported by Steady Health between visits. All respondents strongly agreed or agreed that it was easy and convenient to arrange a visit with their provider in a timely manner, and 23 (92%) would be very or somewhat disappointed if they could not use Steady Health. Of the 23 respondents who were started on a new device or injectable/inhaled medication, 21 (91.3%) strongly agreed or agreed that they had received the training or support they needed.

## Discussion

### Principal Results

This is the first report to our knowledge to explore the implementation of a ground-breaking digital diabetes care model that features universal CGM use among its patients, early and intensive patient engagement by an endocrinologist, and a program of ongoing guidance and accountability to individualized goals between endocrinology visits. Steady Health attempts to address some of the shortcomings of conventional diabetes care by expanding care and support to be continuous rather than episodic, offering telemedicine visits with diabetes specialists without the need for travel or extended wait times, devoting attention to mental health, and encouraging the adoption of transformative diabetes technologies when appropriate. Major innovations include the decoupling of data analysis from visits and a software platform that puts the patient’s daily BG profile into the context of the meals, activities, and therapies that shape it, such that the reviewing clinician has both the time and tools to uncover deeper, less apparent associations within the data. This process of data discovery allows for actionable insights to be presented and discussed with patients over video and messaging to empower them with the knowledge and agency to better manage their diabetes.

The real-world data presented in this analysis suggest that virtual diabetes care that integrates enhanced analysis of CGM and continual close collaboration with endocrinologists and DCESs can be associated with significant improvements in TIR and HbA_1c_ for patients with uncontrolled diabetes. These improvements were notably seen without increasing TBR and were greatest in the patients least able to maintain adequate glycemic control at baseline. Current CGMs provide 96 or 288 BG readings per day, which serve as valuable input for driving behavioral change and guiding treatment decisions, and there is a growing body of literature supporting the use of CGM in patients with type 1 and type 2 diabetes [16-20]. However, not all studies demonstrate meaningful gains [21,22], suggesting that an abundance of BG data alone may not always translate into better glycemic outcomes. Although many of the patients in this study saw a durable improvement in their glycemic control under Steady Health’s care, those who were most engaged with the care team tended to see the greatest improvement overall. The specific interventions that proved successful were diverse and included diet and lifestyle modification, connecting patients with diabetes-focused mental health specialists, premeal bolusing, fine-tuning of insulin dosing (as frequently as daily), and the introduction of new therapies and devices. Importantly, better glycemic control was achieved whether or not an automated insulin delivery system was initiated. Not only did glycemic outcomes improve, but these patients also expressed high levels of satisfaction with the impact of Steady Health’s intervention, the support provided between visits, and the ease of arranging visits with their providers in a timely manner.

The positive findings seen among this cohort of patients also strongly affirm the notion that the education, training, and support for CGM, smart pens, insulin pumps, algorithms, and inhaled insulin can be delivered both safely and effectively in a virtual care setting. A thorough discussion of the risks and benefits of each technology or therapy, an assessment of readiness, and guided instruction on proper use were prerequisites to initiation of each product, and all these interactions were routinely conducted over telemedicine visits. All patients new to CGM were able to self-start with instructional videos and messages, corroborating prior accounts that CGM can be feasibly initiated without in-office training [23]. Most patients also saw improvements in their glycemic control because of these interventions and expressed high levels of satisfaction with the relevant training and support provided by Steady Health.

### Comparison With Prior Work

Another virtual diabetes care model partially incorporating CGM use and clinical support has been previously described [24]: significant improvements in HbA_1c_ were seen among 740 early participants in their telehealth program over a median follow-up period of 4.2 months. The noteworthy differences between these approaches are also relative strengths of this study in that it includes a large proportion of patients with type 1 diabetes, comprehensive CGM use, initiation of smart pens and insulin pumps when appropriate, and the reporting of CGM glycemic outcomes in addition to HbA_1c_. Indeed, an HbA_1c_ value representing lower average glycemia may belie a greater frequency and severity of hypoglycemia, which should not be overlooked, given growing awareness for the short- and long-term consequences of hypoglycemia [25-27]. In this study, most patients were able to achieve greater TIR without increasing TBR, and half achieved the recommended TIR target of 70%.

### Limitations

There were several limitations to this study, including small sample size, self-selection bias, and the lack of a control group. Most of Steady Health’s patients are commercially insured or able and motivated to afford the added cost of CGM and membership. Moreover, because our cohort was relatively young and a majority had type 1 diabetes, these findings may not be generalizable to the greater population of patients with diabetes. There were some patients who saw an initial improvement in glycemic control followed by a deterioration, or vice versa, with subsequent fluctuations over time; arbitrarily defining the comparison end-of-study period as February 2021 did not fully convey the variability in glycemic control seen over longer periods of care. Moreover, the first 4 weeks of available CGM data did not always reflect true baseline glycemic control, as patients were unblinded to their BG readings and often made behavioral changes in response to the patterns seen. Meanwhile, treatment decisions were sometimes made within the first 4 weeks based on clinical judgment to avoid extreme hyperglycemia or hypoglycemia. There were also challenges obtaining a follow-up HbA_1c_ test for many of these patients. Most patients joining Steady Health have a baseline TIR>70% (and a fraction of these patients have prediabetes); their outcomes are not reported here. Longer term data on these and other patients being seen by Steady Health are being collected, and those findings will hopefully be published in the future.

### Conclusions

Patients with uncontrolled diabetes can achieve significant glycemic improvements with a virtual care model that meets them where they are, helps them make the most of their CGM data, and provides continuous multidisciplinary care and support, even between visits.

## Abbreviations

BG: blood glucose
CGM: continuous glucose monitoring
DCES: diabetes care and education specialist
HbA_1c_: hemoglobin A_1c_
TBR: time below range
TIR: time in range
